# The Role of HLA and KIR Immunogenetics in BK Virus Infection after Kidney Transplantation

**DOI:** 10.3390/v12121417

**Published:** 2020-12-09

**Authors:** Marija Burek Kamenaric, Vanja Ivkovic, Ivana Kovacevic Vojtusek, Renata Zunec

**Affiliations:** 1 Tissue Typing Center, Clinical Department of Transfusion Medicine and Transplantation Biology, University Hospital Center Zagreb, 10 000 Zagreb, Croatia; mburek@kbc-zagreb.hr; 2 Department of Nephrology, Hypertension, Dialysis and Transplantation, University Hospital Center Zagreb, 10 000 Zagreb, Croatia; vanja.ivkovic@gmail.com (V.I.); ikovacevicvojtusek@gmail.com (I.K.V.); 3 Department of Public Health, Faculty of Health Studies, University of Rijeka, 51 000 Rijeka, Croatia

**Keywords:** BK virus, BK virus-associated nephropathy, kidney transplantation, human leukocyte antigen, killer-cell immunoglobulin-like receptor, natural killer cells

## Abstract

BK virus (BKV) is a polyomavirus with high seroprevalence in the general population with an unremarkable clinical presentation in healthy people, but a potential for causing serious complications in immunosuppressed transplanted patients. Reactivation or primary infection in kidney allograft recipients may lead to allograft dysfunction and subsequent loss. Currently, there is no widely accepted specific treatment for BKV infection and reduction of immunosuppressive therapy is the mainstay therapy. Given this and the sequential appearance of viruria-viremia-nephropathy, screening and early detection are of utmost importance. There are numerous risk factors associated with BKV infection including genetic factors, among them human leukocyte antigens (HLA) and killer cell immunoglobulin-like receptors (KIR) alleles have been shown to be the strongest so far. Identification of patients at risk for BKV infection would be useful in prevention or early action to reduce morbidity and progression to frank nephropathy. Assessment of risk involving HLA ligands and KIR genotyping of recipients in the pre-transplant or early post-transplant period might be useful in clinical practice. This review summarizes current knowledge of the association between HLA, KIR and BKV infection and potential future directions of research, which might lead to optimal utilization of these genetic markers.

## 1. Introduction

BK virus (BKV) is a small double-stranded DNA virus belonging to the *Polyomaviridae* family. Its genotype consists of a non-coding region, an early coding region (transcribing T antigen), a late coding region (transcribing three viral capsid proteins), and a fourth region which encodes the agnoprotein. The capsid consists of three proteins, VP-1 (the major structural protein), VP-2 and VP-3. BKV was first detected in a renal allograft recipient in 1971 [1,2]. The virus consists of four serotypes marked I, II, III and IV, with serotype I being most prevalent [3]. It is estimated that seroprevalence of BKV is around 80% to 90% [4]. Primary infection (primoinfection) most probably occurs in early childhood via the fecal-oral or respiratory routes and afterwards the virus forms a persistent latent infection of the urothelial (transitional epithelium) and renal tubular cells [2,5,6]. Not much is known on the clinical manifestations of primary BKV infection in non-immunocompromised persons, but it has been shown that seroprevalence in children is around 91% and is reached at 5 to 9 years of age and that it is most likely asymptomatic or presents as an influenza-like illness [7,8]. In the general population, the latent infection normally does not produce any symptoms and reactivation does not occur. However, in the immunocompromised patient BKV infection has a very different course. It has been reported that in the vast majority of renal transplant patients BKV infection follows a clear sequential course of viruria-viremia-nephropathy-allograft loss with viruria present in 30% to 40% and viremia in 10% to 20% of such patients [9]. A study examining donor–recipient BKV genotypes showed a donor origin in viremic kidney allograft recipients [10]. BKV is closely related to two other polyomaviruses, Simian virus 40 (SV40) and JC virus (JCV). In fact, BKV shares 72% of the entire DNA sequence with JCV and 69% with SV40 of JCV is around 50–70% and its primoinfection is asymptomatic, but it can cause serious and frequently fatal infections in immunodeficient and immunosuppressed individuals. In patients with (mostly severe) immunodeficiency, it leads to progressive multifocal leukoencephalopathy (PML), a disease with a frequently dire prognosis characterized by motor deficits, altered consciousness, gait ataxia, and visual disturbances. In renal allograft recipients it can sometimes cause JCV-associated nephropathy (JCVAN), a rare disease which can sometimes lead to allograft loss. SV40 has a seroprevalence of 90% in children and 60% in adults and there is controversial evidence that it might lead to carcinogenesis in humans. However, this link remains to be confirmed. Antibodies to this virus are mostly used as surrogate markers for BKV-associated nephropathy (BKVAN) and JCVAN [4,7].

A gradient rise in serum creatinine is often the only sign of BKVAN aside from detectable viruria and viremia, but if left unchecked, viral replication will ultimately result in interstitial inflammation and fibrosis accompanied by tubular injury [11]. In progredient cases a more severe clinical picture consisting of pyuria and active urinary sediment may arise [12]. Decoy cells in urine might be detected, especially in cases with high viruria and viremia [13]. A diagnosis of BKVAN is histological and both characteristic cytopathic changes (viral inclusions, anisonucleosis, hyperchromasia, polynuclear cells, tubular injury, tubulitis etc.) and positive immunohistochemistry (antibodies against BKV or SV40) are needed [14,15].

Given the importance of early detection of BK viruria and viremia and lack of any definite therapy for BKVAN, it is very important to develop not only the methods for early screening, but also the tools that will enable early (pre-transplant) risk stratification. This will further lead to identification of potential kidney allograft recipients at high risk for BKV reactivation and progression to BKVAN and potential allograft failure and may improve donor-recipient matching and monitoring strategies in these at-risk recipients.

Constitutive factors of the innate immune system play an important role in the defense from numerous viruses, among them natural killer (NK) cells are among the most important. Indeed, human leukocyte antigens (HLA) and killer cell immunoglobulin like receptors (KIR), molecules found on NK cells crucial in recognition and effector arms of innate response, have been shown to have a part in the pathophysiology of BKV infection and to predict and modify the risk for BKVAN [16]. Furthermore, it seems that some specific HLA alleles may be predisposed to BKVAN [17], while some may be protective [18]. In addition to this, KIR genes and KIR receptors, and consequently NK cells have also been implicated in the pathophysiology of BKV infection and nephropathy. It is plausible that HLA and KIR might be factors that could, as mentioned before, enable pre- and post-transplant risk stratification and guide further management in an effort to improve outcomes in allograft recipients.

Preceded by a brief overview of risk factors, screening, treatment and prognosis of BKV infection and associated nephropathy in kidney transplantation, this review aims to summarize the current knowledge on the association of HLA and KIR immunogenetics with BKV infection in renal allograft recipients.

## 2. An Overview of BKV Infection and BKVAN in Kidney Transplantation

### 2.1. Risk Factors, Screening and Treatment of BKV Infection and BKVAN

While a great number of studies examined risk factors for BKV infection, the only meta-analysis published which systematically studied risk factors identified 8 risk factors associated with increased risk for BK viremia (maintenance therapy regimen including tacrolimus, allograft from a deceased donor, recipient of male sex, history of previous transplant, age at transplantation, ureteral stent use, delayed graft function and acute rejection episodes) and two associated with increased risk for BKVAN (maintenance therapy regimen including tacrolimus and acute rejection episodes) [19]. Another meta-analysis specifically examined the risk of therapeutic regimens including mammalian target of rapamycin (mTOR) inhibitors vs. calcineurin inhibitors (CNI) did not find any association of drug regimen with risk of BKV infection [20]. When examining data from individual studies, risk factors for BKVAN can be divided into recipient-, donor- and allograft specific and include: male sex, older age of donor and recipient, cold ischemia time, delayed graft function, episodes of rejection, ureteral stent, use of anti-thymocyte globulin in induction, use of tacrolimus in maintenance therapy, ABO blood group system incompatibility, ischemia/reperfusion injury and recipient or donor seropositivity. Conversely, mTOR inhibitor use has been shown to be a protective factor [9,21,22,23,24].

The current BKVAN therapy protocol consists mainly of immunosuppressive therapy reduction [21]. Moreover, an important point is distinguishing BKVAN from allograft rejection, since the two may present similarly, but are treated in completely opposite ways, i.e., treatment of misdiagnosed rejection in the presence of BKVAN might lead to allograft loss [25,26]. Furthermore, histological surveillance of BKVAN is also problematic from a clinical point of view, which has been thoroughly studied. Menter et al. explored the histopathology of resolving BKVAN which found that this stage is morphologically indistinguishable from interstitial rejection [27]., For an accurate diagnosis it is imperative to obtain adequate and deep samples as BKV has a tropism for renal medulla [28]. Aside from reduction of immunosuppression, which is the only viable treatment strategy, other therapies have been tested and occasionally used. Intravenous immunoglobulins have been shown to be useful in patients who do not respond to the initial reduction of immunosuppression [29] and might lead to additional BKV clearance [30]. A recent study possibly partially elucidated the mechanism behind this, demonstrating that intravenous immunoglobulin administration increases the titer of neutralizing antibodies specific for BKV [30]. On this basis, a very recent proof-of-concept study has stated that intravenous immunoglobulins might be useful in clinical practice and potentially reduce the risk of allograft loss [30]. Some studies showed good results using leflunomide, a prodrug to an antimetabolite A77 1726, usually by replacing mycophenolate mofetil, however, other studies demonstrated conflicting results and their use is controversial [31,32,33]. Cidofovir is an antiviral agent, used also for BKV infection, [34], but its usage is limited due to low efficacy and potential nephrotoxicity [35]. While initial studies showed an effect of quinolone antibiotics [36,37], this was disproven in further, better-designed randomized controlled studies [38,39]. Immunosuppression with everolimus after switching from CNIs were shown to be promising in one retrospective study [40]. Given the limited options of therapy and the established viruria-viremia-nephropathy sequential course, screening aimed at early detection of BK viruria and viremia is of paramount importance. It has been shown that screening for BKV DNAemia enables identification of at least 90% of patients at risk before significant repercussions for the allograft. However, quantitative nucleic acid testing (NAT) is still underutilized [41,42]. It has been demonstrated that most cases of BKVAN occur in the first 6 months or 1-year post-transplant and recent research showed that only around 20% to 30% of BKV DNAemia events occur later than 6 months post-transplant, which strongly points to the need for strict early surveillance [43,44,45]. Several screening and intervention strategies have been developed, based on testing frequency and detection method. Kidney Disease Improving Global Outcomes (KDIGO) guidelines for kidney transplant recipients suggest screening all recipients of BKV using quantitative plasma NAT at least monthly for the first 3 to 6 months after transplantation, then every 3 months until the end of the first post-transplant year, and subsequently whenever there is an unexplained rise in serum creatinine and after treatment for acute rejection [46]. American Society of Transplantation (AST) guidelines recommend quantitative NAT as the main testing method to be performed monthly up to month 9 post-transplant followed by testing every 3 months up to 2 years post-transplant or at the time of surveillance and indication of an allograft biopsy. Stepwise reduction of immunosuppressive medications is recommended when BKV plasma NAT is persistently (for 3 weeks and longer) greater than 1000 copies per milliliter (mL) [47]. Complementary to quantitative polymerase chain reaction (qPCR) DNA testing, several institutions also use other methods, such as urine cytology (decoy cells) and virus RNA [46]. Nankivell et al. found that, although decoy cells detection have a high specificity and negative predictive value for BKVAN, quantitative viremia determination by qPCR was superior having high sensitivity, specificity and negative predictive value [48]. However, urine cytology, especially quantification of decoy cells, might be a useful additional tool in experienced centers especially in situations when kidney biopsy cannot be performed. Given a recent study on the need for deep sampling during kidney biopsy for obtaining an adequate tissue sample, urine cytology might be useful in cases where no or scarce medulla was obtained [28]. Looking for an integrated method of surveillance, a review by Comoli et al. summarized the current knowledge on BKV-specific cellular immunity, found that it is associated with viral clearance and that prospective monitoring for viremia coupled with specific immunity and B-cell alloimmune surveillance might lead to prevention and better outcomes in BKVAN [49].

### 2.2. Prognosis of BKV Infection and BKVAN

One of the first important studies focused on outcomes in patients with BKVAN found that in a cohort of 1001 renal and renal/pancreas allograft recipients, 41 patients developed BKVAN with median of 318 days to diagnosis and that allograft survival at 6 months, 1, 3, and 5 years was 97%, 90%, 58%, and 47%, respectively, compared to a contemporaneous cohort of patients without BKVAN which had significantly better 6 months, 1, 3 and 5 year allograft survival (94%, 92%, 83%, and 76%, respectively). Allograft loss occurred in 46% of subjects and BKVAN diagnosis was preceded with a steep fall in estimated glomerular filtration rate (eGFR) [50]. A small Chinese prospective study found that 5.6% of patients developed BKVAN in the first year post-transplant and that a reduction in immunosuppression led to resolution in all patients [51]. In a study of 213 kidney transplant recipients, high BK viremia (defined as (≥10^4^ copies/mL) occurred in 17.4% and low viremia occurred in 49.3% of patients, while BKVAN occurred in 4.2% of patients [52]. In another retrospective long-term study (mean follow up of over 7.7 years), the incidence of BKVAN was 4.0% and 60.0% of patients with BKVAN and acute rejection progressed to allograft loss [53]. In patients sequentially monitored for BK viruria and viremia in which the CNI dose was reduced first when sustained BK viremia occurred (>1000 copies/mL) at median follow up of 5 years, 11% of patients developed rejection [43]. A report that included 609 patients of which 130 developed BK viremia during the first post-transplant year and who were then classified as transient low viremia, transient high viremia, persistent low viremia, and persistent high viremia (based on BK viral load cut-off of 10,000 copies/mL and infection duration cut-off of 3 months) demonstrated that low viremia (either transient or persistent) did not affect long-term outcomes. Contrary to this, persistent high viremia was associated with higher risk for BKVAN and subsequent allograft dysfunction and transient high viremia was associated with worse long-term allograft function [54]. An analysis of outcomes of Chinese renal allograft recipients treated for BKVAN showed that after a mean follow up of 14.4 months, BK viruria disappeared in 19.5% and BK viremia in 90.2%. One-year graft survival was excellent, while 5-year graft survival was 85.7% [55]. In a study of 1904 renal allograft recipients of which 17.3% had BK viremia and 3.6% had BKVAN, high serum creatinine, early BKVAN (defined as occurring within 6 months post-transplant) and microvascular inflammation were independently associated with higher risk of allograft loss [56].

Re-transplantation after allograft loss due to BKVAN is considered safe after complete resolution of viremia and is frequently performed, especially considering that most BKV infection is donor-derived. A very recent study showed that there were no differences in death-censored graft survival, acute rejection episodes and patient survival between patients who underwent re-transplantation after first allograft loss due to BKVAN versus other causes [57].

## 3. Association of HLA with BKV Infection and BKVAN

The HLA region is a cluster of highly polymorphic genes representing the major histocompatibility complex (MHC) in humans [58]. HLA molecules, encoded by HLA class I and HLA class II genes, are cell-surface glycoproteins that present intracellular and extracellular peptides to T cells [59]. Viral peptides presented by HLA class I molecules interact with CD8-positive T-cells while HLA class II molecules interact with CD4-positive T-cells [60]. In this way, HLA molecules are involved in cellular and humoral adaptive immune responses and have a major role in the control of infection and inducing anti-viral immune response. It has been shown that certain HLA alleles provide protection or lead to slower progression of viral infection by preferential presentation of epitopes from conserved viral proteins to immune system [61,62,63]. Therefore, a number of studies tried to identify the influence of donor or recipient HLA alleles and other HLA-related risk factors (number of HLA mismatches, percent of panel reactive antibodies, HLA donor specific antibodies) in the development of BKV infection and BKVAN (Table 1).

### 3.1. HLA Alleles

Several studies have demonstrated that specific HLA alleles might be associated with risk of BK viremia and that some alleles confer increased risk, while some are protective. One of the most probable independent risk factors for sustained BKV viremia and BKVAN could be the HLA-C*07 allele i.e., HLA-Cw7 antigen. Bohl et al. reported that the absence of an HLA-Cw7 in either the donor or recipient was associated with a higher rate of BKV replication. Although they could not clearly separate the relative influences of the donor’s and recipient’s HLA-Cw7, they suggested that lack of HLA-Cw7 in donors might have a stronger association with BKVAN [64]. A very interesting case series by Gheith et al. explored the outcomes in 5 recipients with allografts received from BKV positive donors. Interestingly, HLA-Cw7 was present in 4 recipients, and in the fifth case, this antigen was present in the donor. At the last follow-up (mean of 21.6 months), there were no cases of BKV infection. While the evidence was weak, the authors postulated that HLA-Cw7 might have a protective role from BKVAN [66]. Finally, a recent study from our group showed that lower proportion of recipients in BKVAN group had a positive HLA-C*07 donor, confirming that donor HLA-C*07 positivity was significantly associated with lower odds for BKVAN [67]. The exact mechanism of action remains unknown although it is suggested this effect could be due to the HLA-Cw7 antigen involvement in BK antigen presentation and initiating a cytotoxic T-cell or NK cell immune mediated response [64].

Masutani et al. demonstrated that some alleles might be protective and concluded that HLA-A2, -B44 and -DR15 decreased the risk of developing BK viremia [18]. Teutsch et al. found that the presence of HLA-A9 in the donor and HLA-A2 in the recipient showed a significant association with the development of polyomavirus-associated nephropathy while recipients positive for HLA-A28 and HLA-A68 showed a higher risk for the development of viremia but not for progression to nephropathy [65]. Dogan et al. showed that matching for HLA-A24 and -B55 subgroups was an independent risk factor for BK viremia and that HLA-A24 was also a predictor of BK viremia (defined as ≥10,000 copies/mL) [17].

A recent retrospective study by Kavuzlu et al. investigated the interrelationship of HLA-A, -B and -DR with BKV in renal transplant recipients and found that HLA-B*13 allele is protective, conferring an 86% odds reduction for BKV infection. Conversely, HLA-DRB1*03 allele increased odds for BKV infection 2.5-fold [69]. Wunderink et al. have studied the potential association of HLA class I and II antigens with risk of BKVAN in Dutch renal allograft recipients and have demonstrated that HLA-B51 recipient positivity reduces the risk of post-transplant BK viremia approximately fivefold. While the difference was not significant, possibly because of only 12 instances of BKVAN in the whole study population, it is important to note that BKVAN was not diagnosed in any of the HLA-B51–positive recipients, i.e., that all cases of BKVAN were in HLA-B51-negative recipients [63].

When looking at epitopes, a study by Roark et al. reported the HLA-DQB1 epitope, EV-RGI84-90, (common to DQ5/DQ6) to be significantly associated with resistance to BKV reactivation in renal transplant recipients and additionally, renal transplant recipients homozygous for DQ5/DQ6 had greater resistance to BKV in contrast to recipients homozygous for DQ2/DQ3/DQ4, who were at higher risk for disease. They concluded that presentation of BKV peptides on HLA-DQ5/DQ6 to T cells may be required to prevent BKV reactivation [70].

There is also a role for non-classical MHC antigens in BKVAN. Rohn et al. have shown that in a cohort of 278 living-donor–recipient pairs HLA-E, a known regulator of anti-viral immunity previously demonstrated to bind and present antigenic peptides from several viruses, including cytomegalovirus (CMV), is associated with risk of BKV infection [72,84,85]. Namely, recipient homozygosity for the HLA-E*01:01 allele had increased susceptibility to BKVAN. Interestingly, a previous study reported that recipient presence of another allele, HLA-E*01:03, is associated with risk of CMV infection in living-donor kidney allograft recipients [86]. The same group has also shown that HLA-G 3’UTR-4 haplotype positivity in donors and recipients is associated with BKV replication or nephropathy making HLA-G 3’UTR variants as predisposition markers for detecting BKV infectious complication or rejection-susceptible recipients prior to kidney transplantation [73].

Aside from MHC, a role was also postulated for MHC class I polypeptide–related sequence A (MICA) as one study reported that a lower incidence of BKV reactivation was noted in recipients of renal allografts from donors carrying mutated MICA A5.1 and that a donor/recipient mismatch for MICA A5.1 is crucial for BKV reactivation and development of BKVAN [74].

### 3.2. HLA Mismatch, HLA and ABO Blood Group System Incompatibility and Desensitization

Several studies examined the association with allele-specific and overall HLA mismatch and BK viremia or BKVAN. The association of BKVAN with increased HLA mismatching was first observed by Hirsch et al. [75] and later confirmed in the study of Awadalla et al. [76]. An observational study by Favi et al. showed that there was a higher proportion of patients with >4 HLA mismatches in patients with detectable BK viremia ≥1000 copies per mL (26.7% vs. 13.4%). Moreover, multivariate analysis which included a number of demographic and clinical parameters showed that >4 HLA mismatches are independently associated with a risk of BK viremia in kidney transplant recipients [78]. In a study of Hässig et al., acute rejection episodes and number of HLA mismatches were the strongest independent predictors of BK viremia [16]. However, as demonstrated by Helanterä et al., BKVAN can develop even in low-risk populations (defined in their case as HLA well-matched population on low-dose cyclosporine-based triple maintenance immunosuppressive therapy) as 9% of the subjects in their study had BK viremia, while 4% developed BKVAN [87]. These results further accentuate the importance of efficient monitoring strategies aimed at early detection of viruria and viremia.

Sharif et al. confirmed that ABO incompatible patients are at higher risk of BKVAN and that a subset of patients who did not exhibit a specific graft-accommodation phenotype might be at a heightened risk [24]. Conversely, Kwon et al. did not find a difference in the incidence of BKVAN between crossmatch-positive patients with and without ABO incompatibility [88]. Interestingly, when looking at HLA incompatibility, a small study showed that even after a desensitization protocol BK viremia occurred in one patient (4%) [89]. Furthermore, another study reported that allograft recipients who underwent desensitization for ABO incompatibility or HLA mismatch had a higher incidence of BKVAN [90].

### 3.3. HLA Panel Reactive Antibodies

The degree of sensitization to HLA antigens expressed as calculated panel reactive antibody (cPRA) could predispose to BKV because of more intensive induction and maintenance immunosuppression used in these patients or through a rejection-related inflammatory process, which increases the chance of BKV reactivation [68]. Several studies [18,68,76,77] explored the association of cPRA with BKV activation and all concluded that the cPRA percentage is not a risk factor for BKV or BKVAN. Only Borni-Duval et al. found that cPRA > 0% was associated with higher risk of BKV (compared to cPRA = 0%), but statistical significance of cPRA was lost in multivariate analyses [79]. A retrospective study by Al-Husseini et al. explored the association of HLA and cPRA with BKV activation (defined as BK viremia >10,000 copies per mL) and found that cPRA, individual HLA antigens and HLA antigen matching were not associated with BK viremia. However, the study included only approximately 25% patients with cPRA of 20% or higher and only around 18% BKV positive patients [68].

Contrary to these findings, a study by Parajuli et al. found that high PRA (defined as PRA > 80% vs. 80% or lower) was and independent protective factor against viral infection (defined as BKV infection or CMV disease) [91].

### 3.4. HLA Donor-Specific Antibodies

Patel et al. examined the association of de novo donor-HLA specific antibodies (DSA) and BK viremia, and specifically if there is a link between these two events, i.e., formation of de novo DSA and occurrence of BK viremia. A total of 28% of initially DSA-negative patients became DSA-positive after BK viremia compared with 26% of patients which did not have BK viremia (a non-significant difference). However, a higher number of HLA mismatches was an independent predictor of BK viremia [82]. Similarly, Dieplinger et al. found that BK viremia is independently associated with higher rates of de novo DSA [80]. Everly et al. found that in a cohort of African American allograft recipients, BK viremia was one of the factors associated with development of de novo DSA (occurring in the first 24 months after transplantation) [83]. A pivotal study by Sawinski showed that persistent BK viremia (defined as lasting at least 140 days) was strongly associated with the development of class II, but not class I de novo DSAs [81].

## 4. Duality of NK Cells in Kidney Transplantation

Administration of immunosuppressants after transplantation targets the adaptive immune system thus increasing the importance of the innate immune system with NK cells as one of the most important protagonists of immune response against viral infections. In addition to their well-known role in anti-viral and anti-tumor defense, there is evidence that NK cells have a very important role in the pathophysiology of antibody-mediated rejection (ABMR) and graft failure after kidney, heart, lung and liver transplantation [92,93,94,95,96]. The mechanism and regulation of NK cell activation consist of a balance between activating and inhibitory signals from a large number of diverse NK cell receptor gene families on the NK cell surface including KIRs, killer cell lectin-like receptors (LILR), leukocyte immunoglobulin-like receptors (LAIR), and natural cytotoxicity receptors (NCR) [97,98]. In transplantation, NK cell activation and respond against the allograft can occur through different mechanisms: detection of missing-self HLA class I molecules, antibody-dependent cell-mediated cytotoxicity (ADCC), detection of stress molecules and inflammatory environment [99,100].

### 4.1. Role of NK Cells in ABMR Mediated through Fc Receptor (CD16)

As shown by biopsy findings of microcirculation damage and C4d deposition in peritubular capillaries, occurrence of DSAs against mismatched donor HLA antigens plays the central role in ABMR. However, Hidalgo et al. found dominant NK cell transcripts in kidney biopsies from patients with ABMR and a subsequent study by Yazdani et al. used microarray transcriptomic data to identify genes that are differentially expressed in ABMR and reported that NK cells also play an important role in the pathophysiology of ABMR and graft failure after kidney transplantation [92,101]. In healthy kidneys, NK cells maintain barrier integrity and protect against pathogens. Turnet et al. proposed a mechanism for a pathogenic role of NK cells in ABMR through the expression of FcγRIII (CD16) [102]. Binding of DSA to allograft endothelial cells and interaction with the Fc receptor CD16 on NK cells trigger ADCC directed against endothelial cells, resulting in microvascular injury of the kidney allograft. Furthermore, infiltration of activated NK cell into the graft can discriminate ABMR from T-cell–mediated rejection (TCMR) in biopsy samples and can predict graft failure after transplantation [92].

### 4.2. Role of NK Cells in Viral Infections Mediated through KIRs

Apart from antibody-dependent NK cell activation, antibody-independent NK cell activation through non-self or altered-self and/or missing-self HLA class I ligands on target cells are two main control mechanisms of circulating NK cells. In the missing-self hypothesis, the absence of HLA class I molecules on target cells leads to the loss of inhibitory KIR receptor/HLA ligand interactions and subsequently to activation of NK cells. Downregulation of HLA class I molecules is an escape mechanism from T-cell-mediated responses of many viruses and tumor cells, but this leaves them “visible” to NK cell attack through missing-self recognition. In the non-self or altered-self mechanism, activating receptors recognize viral or endothelial stress proteins directly or presented on HLA class I molecules. 

The main mediators of NK cell alloreactivity are KIR receptors. A growing number of studies have documented associations of KIR genotype with different viral infections asserting KIR3DS1 as the most studied activating KIR (aKIR) in the setting of infectious diseases. In patients infected with human immunodeficiency virus (HIV) genetic combination *KIR3DS1*/HLA-Bw4-80I was associated with slower disease progression while the presence of the *KIR3DL1* gene with HLA-B*57 was associated with slowed disease progression and also induced a protective effect against HIV acquisition [103,104]. A review by Hölzemer at al. provides a detailed evaluation of the interactions of KIRs with classical and non-classical HLA class I molecules in HIV infection [105].

Research of CMV infection following solid organ transplantation demonstrated that transplant recipients should be resistant to CMV infection if they carried KIR B-haplotype [106] and particularly if they carried a telomeric region of KIR B haplotype (specifically genes *KIR2DL5, KIR2DS1, KIR2DS5* and *KIR3DS1*) [107,108]. Furthermore, NK cells expressing *KIR3DL1* were associated with CMV reactivation [109]. Gonzalez et al. analyzed incidence of CMV viremia in patients according to the type of transplanted organ and found a protective effect of the KIR haplotype Bx in patients receiving lung or heart allografts, whereas no such association was found in kidney or liver allografts [110].

Several studies identified an association of KIR genes or haplotype with a risk of viral infections, including hepatitis B [111], hepatitis C [112], influenza [113], Ebola [114], herpes simplex virus [115,116], Epstein–Barr virus [115,117], varicella zoster [115], human papilloma virus [118]. However, direct mechanistic evidence for a role of aKIRs in recognition of virus-infected cells is still missing.

### 4.3. Role of KIR-HLA Interactions on NK Cell Responsiveness in ABMR

**Figure 1:**
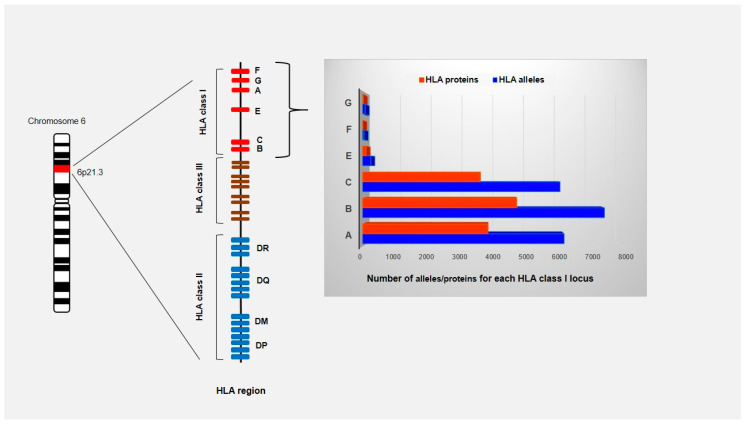
Schematic representation of HLA region located on the short arm of chromosome 6 (6p21.1 to p21.3). The HLA gene complex is subdivided into class I, class II and class III regions based on the structure and function of the molecules that these genes encode. The HLA class I and class II genes encode molecules that bind and present peptide fragments while the class III region contains genes for cytokines and the components of the complement system. HLA genes are codominant expressed and highly polymorphic with 25,958 allele sequencies known to date (IPD-IMGT/HLA Database on 1 October 2020). Only the number of HLA class I alleles and proteins are shown since they are ligands for KIRs.

**Figure 2:**
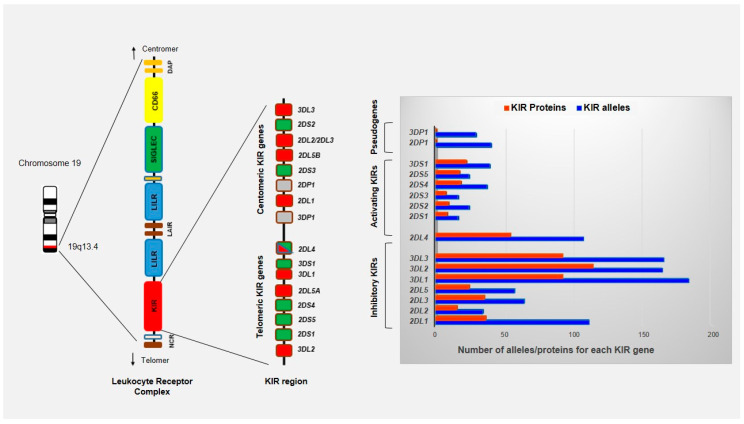
Schematic representation of KIR region located on long arm of chromosome 19 (19q13.4) within the leukocyte receptor complex (LRC). The LRC complex constitutes a large cluster of genes that encode proteins containing immunoglobulin-like domains (NCR, KIR, LILR, and LAIR), signaling proteins (SIGLECs) and CD66 family proteins. A total of 14 functional KIR genes and 2 pseudogenes have been identified and organized into two broad haplotypes termed A and B. KIR genes of one haplotype are divided into centromeric and telomeric regions by the framework genes *KIR3DL2*, *KIR2DL4*, *KIR3DP1* and *KIR3DL3*. KIR genes are highly variable with regard to both gene content and allelic polymorphism with 1110 alleles known to date (IPD-KIR Database on 1 October 2020).

KIRs and NKG2A inhibitory receptors specific for self HLA-class I molecules determine whether NK cells will kill their target cells or remain inactive [97]. During the process of NK cell “licensing” or “education” only NK cells expressing at least one inhibitory receptor for self HLA-class I antigens will achieve maturation while all others are deleted or stay anergic [119,120]. Since HLA and KIR are two of the most polymorphic genetic systems in humans (Figure 1 and Figure 2), this polymorphic diversity has a significant impact on KIR-HLA binding strength and variation in binding specificity, which quantitatively influence licensing, strength of signaling, and ADCC [121]. Parsons et al. demonstrated that general levels of ADCC mediated against target cells were significantly higher in a group of HLA-Bw4(+)KIR3DL1(+) individuals than in HLA-Bw4(−) group and thus indicate a prominent role for KIR3DL1/HLA-Bw4 interactions in licensing NK cells for CD16-mediated effector function [122]. In a subsequent study they also demonstrated that NK cells educated through 3DL1/HLA-Bw4 interactions mediate the potentially beneficial anti-HIV ADCC responses against autologous target cells, but also stress the possibility of KIR3DL1-educated NK cells that in the presence of autoantibodies or uninfected cells carrying viral proteins could lead to immunopathology [123].

## 5. Role of KIR and Prognostic Implications of KIR Type in BKV and BKVAN

The KIRs are the most polymorphic NK cell receptors which are encoded with 14 functional KIR genes and two pseudogenes. Depending on the structure of their cytoplasmic tail, KIR receptors can induce an inhibitory (KIR2DL1, KIR2DL2, KIR2DL3, KIR2DL5, KIR3DL1, KIR3DL2 and KIR3DL3) or activating signal (KIR2DS1, KIR2DS2, KIR2DS3, KIR2DS4, KIR2DS5 and KIR3DS1) or both (KIR2DL4). The main ligands for most KIR receptors are classical or non-classical HLA class I molecules (HLA-C molecules with asparagine (group C1) or lysine (group C2) at position 80; HLA-A and HLA-B molecules that carry a Bw4 epitope; HLA-A3/A11; HLA-G; HLA-F). Ligands for the majority of activating KIRs are still poorly understood and possible candidate ligands might be some non-HLA proteins such as tumor specific antigens, stress ligands on a damaged target cells (e.g., MICA, ULBP1, PVR, Nectin-2, CD48) or foreign antigens expressed on infected cells [124]. Based on different gene content, KIR genes segregate as haplotype A (conserved set of six inhibitory and one single activating KIR gene) and haplotype B (variable content and may contain up to 5 additional activating receptors). Each KIR gene cluster of one haplotype can be divided into two haplotype blocks: centromeric (Cen A or Cen B) and telomeric (Tel A or Tel B).

Although there are very limited data, it is very likely that NK cells also have a role in controlling BKV infection (Table 2). Trydzenskaya et al. confirmed this assumption and demonstrated that genetic predisposition to BKV infection and BKVAN depends on NK cell levels [125]. They found that patients with BKVAN had a low number of activating KIRs, particularly lower frequencies of *KIR3DS1*, and consequently proposed a protective role of the Tel B KIR haplotype for BKV reactivation and BKVAN. Lower number of activating KIRs among patients with BKVAN was later confirmed in our study [67] but no difference in any single particular aKIR gene frequency was observed. In addition, we confirmed that the KIR AA haplotype was more frequent in BKVAN group. We did not find an effect of KIR haplotype B in replication and protection from BKV which is consistent with data from Schmied et al. [115]. Also, according to data from Trotter et al., no influence of *KIR2DS4* polymorphisms in kidney transplant recipients to BK viremia or acute rejection was proven [126]. Brochot et al. did not observe an impact of KIR haplotype or number of aKIR on the incidence of BKV replication [127]. Their results suggested that *KIR2DS1*+/non-homozygous HLA-C2 recipient and HLA-C2+ donor, and *KIR2DS2*+/non-homozygous HLA-C1 recipient and HLA-C1+ donor are associated with BKV reactivation after kidney transplantation.

## 6. HLA, KIR and BKV

**Figure 3:**
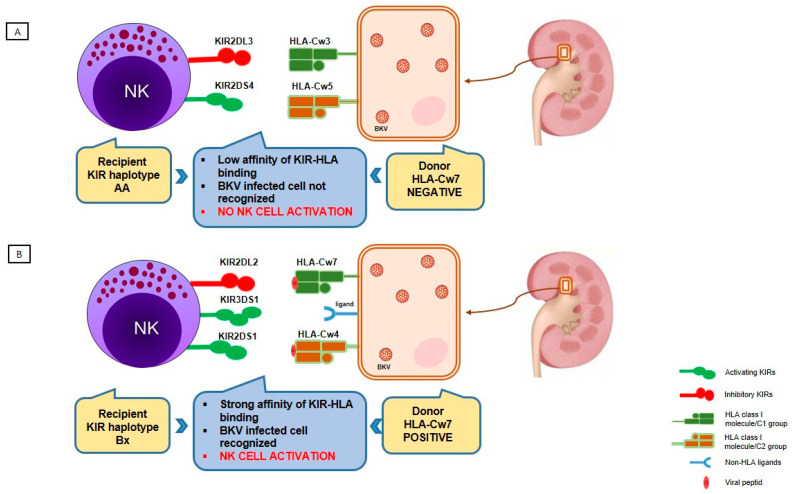
Proposed combined donor HLA genotype and recipient KIR haplotype effect on controlling BKV infection in kidney transplant recipients and further effect on BKV-associated nephropathy development. (**A**) Lack of an HLA-Cw7 molecule in donor causes non-efficient binding and presentation of viral peptides to NK cells in combination with low affinity of KIR-HLA binding; conversely, recipient carrying KIR haplotype AA lacks activating KIR receptors, which could recognize other non-HLA ligands to activate the NK cell. Without NK cell activation, a virus-infected cell remains unrecognized. (**B**) HLA-Cw7 molecule in donor strongly binds the viral peptide and presents it to recipient’s NK cells carrying KIR receptors of haplotype Bx, which have strong affinity for HLA ligands; recipients carrying KIR haplotype Bx have higher number of activating KIRs leading to NK cell activation and the killing of the infected cell.

High polymorphism and independent segregation of HLA and KIR genes on chromosomes 6p21.3 and 19q13.4, respectively cause a wide range of different KIR-HLA ligand combinations in each individual which is of particular importance at the population level as it helps the immune system to adapt to a wide variety of infectious pathogens [124]. In an effort to understand the role of the HLA and KIR systems in viral infections, it is necessary to look at the combined effect of KIR-HLA interactions that might differ from what would be expected from each arm separately. The aforementioned studies examined the distinct relevance of KIR and HLA molecules in the immune response against BKV. We recently reported data of the effect of donor HLA genotype/recipient KIR haplotype on the risk for BKVAN and showed that donor HLA-C*07 negative status taken together with recipient KIR haplotype AA and donor HLA-C*07 negative status taken together with lack of aKIR in recipients were significantly more frequent in the BKVAN group [67]. These results and other similar ones can potentially be explained based on a study by Calap et al. examining the genetic immune escape mechanism of BKV which is associated with HLA-C epitopes [128]. Namely, they predicted the peptide-binding affinities of potential HLA class I epitopes and found three HLA-C epitopes that might be specifically involved in the immune response against BKV through its peptide selection capacity for viral peptides. Therefore, hypothetically, the lack of an HLA-C molecule with appropriate epitopes will not bind efficiently and will not present the viral peptide to the immune system (Figure 3). Furthermore, due to the lack of aKIRs, it will not be able to activate NK cells and the virus-infected cells will not be recognized. It is possible that the observed association of other HLA loci with BKV can be explained with HLA-C molecules, but in the majority of studies HLA-C typing data was not available and analysis could not be performed.

## 7. Future Directions of Research

From a clinical point of view, BKVAN is still a significant problem in kidney transplantation, even in the era of improved screening and early detection strategies, which ultimately leads to a minimization of immunosuppressive therapy. The aforementioned strategy leads to patient under-immunosuppression and, even in the scenario of successful viral load reduction or stabilization of graft function in developed BKVAN, new clinical challenges emerge. These include de-novo DSA production and complications related to histological surveillance of allograft biopsies. Therefore, a new concept based on the assessment of these genetic risk factors and aimed at detecting recipients prone to BKV infection might offer a new perspective in recipient–donor matching. This approach has to be widely tested in prospective studies assessing KIR and HLA recipient and donor status in large cohorts of allograft recipients.

Future research analysing BKV infection in recipients undergoing re-transplantation with at-risk donor/recipient HLA-C combinations would clarify their potential predictive status, but this association might be confounded by different induction immunosuppression protocols used in re-transplantation.

Additional efforts should be made to assess the influence of different immunosuppression types in these cases. It would be very interesting to test a potential additional beneficial effect of m-TOR inhibitors in cases with a potentially protective combination of donor/recipient HLA and KIR status.

Finally, the complex duality of NK cells in ABMR and cytotoxic effects on viral infected cells which are both mediated via ADCC should also be examined in the scenario of BK viral infection and ABMR.

## 8. Conclusions

BKV infection in kidney transplant recipients carries a great risk for graft function deterioration or loss. HLA donor and recipient status, NK cell function, and their KIR status influence the risk of BKV infection especially in different recipient–donor KIR-HLA matches that might prove to be clinically useful in determining the patient at risk either pre- or early post-transplantation which might lead to personalized monitoring and early detection strategies and could, overall, improve patient and allograft outcomes. Further studies are needed to elucidate and quantify this risk.

## Figures and Tables

**Table 1:** Chronological list of studies demonstrating protective and/or deleterious effect of human leukocyte antigens (HLA) alleles and HLA-related factors to BK viremia and/or risk for BKV-associated nephropathy (BKVAN).

References	Factor	n	Clinical Effect
	**HLA Class I Allele** **(Classical)**		
Bohl et al., 2005 [64]	Cw7 neg	195	In D; in R; in both D and R— **↑** BK viremia and BKVAN
Masutani et al., 2013 [18]	A2 pos B44 pos DR15 pos	998	In R—**↓** BK viremia In R—**↓** BK viremia In R—**↓** BK viremia
Teutsch et al., 2015 [65]	A9 pos A2 pos A28/A68 pos	329	In D—**↑** BKVAN In R—**↑** BKVAN In R—**↑** BK viremia
Gheith et al., 2015 [66]	Cw7 neg	5	In D; in R—**↑** BK viremia
Dogan et al., 2017 [17]	A24 pos B55 pos	183	R and D matched—**↑** BK viremia R and D matched—**↑** BK viremia
Wunderink et al., 2018 [63]	B51 pos	407	In R—**↓** BK viremia and BKVAN
Kovacevic Vojtusek et al., 2019 [67]	C*07 pos	23	In D—**↓** BKVAN
El Husseini et al., 2019 [68]	HLA-A, -B, -C		No association
Kavuzlu et al., 2020 [69]	DRB1*03 pos B*13 pos	232	In R—**↑** BKV infection In R—**↓** BKV infection
	**HLA Class II Allele**		
Roark et al., 2016 [70]	DQ5/DQ6 pos	102	In R—**↓** BKV viremia In D—no association
Shah et al., 2016 [71]	DQ2/DQ3/DQ4 pos DQ5/DQ6 pos	433	In R—**↑** BK viremia In R—**↓** BK viremia
	**HLA Class I Allele** **(Non-Classical)**		
Rohn et al., 2019 [72]	HLA-E*01:01 pos HLA-E*01:01 homozygote	278	In R—**↓** BKVAN In R—**↓** BKVAN
Rohn et al., 2019 [73]	HLA-G 3’UTR-4 haplotype pos	251	In R and D— **↑** viremia and BKVAN
Tonnerre et al., 2016 [74]	MICA A5.1 pos	144	In D—**↓** BKVAN D/R MM **↑** BKVAN
	**HLA MM**		
Hirsch et al., 2002 [75]	MM 3–6	78	**↑** viremia and BKVAN
Awadalla et al., 2004 [76]	MM > 4	40	**↑** BKVAN
Bohl et al., 2005 [64]	MM 0–6	195	No association for BK viremia
Hässig et al., 2014 [16]	MM > 4	152	**↑** BK viremia
Suleiman 2017 [77]	MM 0–2; 3–4 or 5–6	537	No association for BK viremia
Favi et al., 2019 [78]	MM > 4	629	**↑** BK viremia
El Husseini 2019 [68]	MM 0–2; 3–4 or 5–6	649	No association for BK viremia
Kavuzlu et al., 2020 [69]	MM 0–6	232	No association for BK viremia
	**cPRA**		
Awadalla 2004 [76]	cPRAneg; cPRA > 0%	40	No association
Borni-Duval et al., 2013 [79]	Cpra > 0%	240	**↑** BK viremia and BKVAN
Masutani et al., 2013 [18]	cPRAneg; cPRA > 0%	998	No association
Suleiman 2017 [77]	cPRAneg; cPRA > 0%	537	No association
El-Husseini 2019 [68]	cPRAneg; cPRA > 0%	649	No association
	**HLA DSA**		
Dieplinger et al., 2015 [80]		174	BK viremia associated with higher rate de novo DSA
Sawinski et al., 2015 [81]		785	persistent BK viremia associated with class II de novo DSA
Patel et al., 2016 [82]		1019	No association of de novo DSA and BK viremia
Everly et al., 2017 [83]		341	BK viremia associated with higher rate of de novo DSA

BKVAN—BK virus-associated nephropathy; cPRA—calculated panel reactive antibody; D-donor; DSA—donor-specific antibody; MM—mismatch; n—number of recipients/donors included in the study; neg—negative; R—recipient; pos—positive; **↑**—increased risk for; **↓**—decreased risk for.

**Table 2:** List of studies demonstrating protective and/or deleterious effect of KIR genes/receptors to BK viremia and/or risk for BKVAN.

References	Factor	n	Clinical Effect
Trydzenskaya et al., 2013 [125]	Low number of aKIR *KIR3DS1* neg Tel B KIR haplotype	48	In R—**↑** BKVAN In R—**↑** BKVAN In R—↓ BK viremia and BKVAN
Brochot et al., 2016 [127]	KIR haplotype aKIR number R:*KIR2DS1*/C1C2 + D:C2 R:*KIR2DS2*/C1C2 + D:C1	103	No association with BK viremia No association with BK viremia **↑** BKV reactivation **↑** BKV reactivation
Trotter et al., 2018 [126]	*KIR2DS4*	1010	No association with BK viremia
Kovacevic Vojtusek et al., 2019 [67]	Lacking aKIR KIR haplotype AA R:KIR AA pos + D:Cw7 neg	23	In R—**↑** BKVAN **↑** BKVAN **↑** BKVAN

aKIR—activating KIR receptors; BKV—BK virus; BKVAN—BK virus-associated nephropathy; C1—HLA-C KIR ligands group 1; C2—HLA-C KIR ligands group 2; D—donor; n—number of recipients/donors included in the study; neg—negative; R—recipient; ****↑****—increased risk for; ****↓****—decreased risk for.
